# European perspectives on efforts to reduce antimicrobial usage in food animal production

**DOI:** 10.1186/s13620-019-0154-4

**Published:** 2020-01-27

**Authors:** Simon J. More

**Affiliations:** Centre for Veterinary Epidemiology and Risk Analysis, UCD School of Veterinary Medicine, University College Dublin, Belfield, Dublin, D04 W6F6 Ireland

**Keywords:** Antimicrobials, Usage, Resistance, Food animals, Europe

## Abstract

New regulations on veterinary medicines and medicated feed will substantially influence antimicrobial prescribing and usage throughout Europe into the future. These regulations have been informed by a very large body of work, including the substantial progress towards reduced antimicrobial usage in food animal production in a number of member states of the European Union (EU). This paper seeks to summarise European perspectives on efforts to reduce antimicrobial usage in food animal production. Work within the EU is informed by the global action plan of the World Health Organization, which includes a strategic objective to optimise the use of antimicrobial medicines in human and animal health. There is ongoing measurement of trends in antimicrobial usage and resistance throughout the EU, and detailed information on strategies to reduce the need to use antimicrobials in food animal production. Substantial scientific progress has been made on the measurement of antimicrobial usage, including at herd-level, and on the objective assessment of farm biosecurity. In a number of EU member states, monitoring systems for usage are well-established, allowing benchmarking for veterinarians and farms, and monitoring of national and industry-level trends. Several countries have introduced restrictions on antimicrobial prescribing and usage, including strategies to limit conflicts of interest around antimicrobial prescribing and usage. Further, a broad range of measures are being used across member states to reduce the need for antimicrobial usage in food animal production, focusing both at farm level and nationally. Veterinarians play a central role in the reduction of antimicrobial usage in farm animals. Ireland’s National Action Plan on Antimicrobial Resistance 2017–20 (*i*NAP) provides an overview of Ireland’s commitment to the development and implementation of a holistic, cross-sectoral ‘One Health’ approach to the problem of antimicrobial resistance. The new regulations offer an important springboard for further progress, in order to preserve the efficacy of existing antimicrobials, which are a critical international resource.

## Introduction

**Fig. 1:**
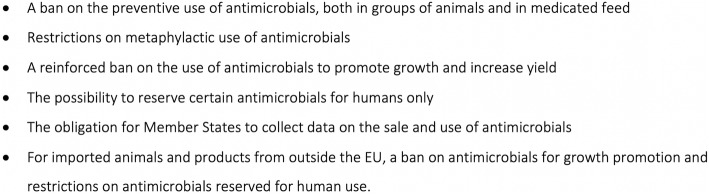
New measures to fight antimicrobial resistance, as outlined in Regulation (EU) 2019/6 (veterinary medicines) and (EU) 2019/4 (medicated feed) [1, 2]. These regulations will enter into force within the European Union from 28 January 2022

New regulations on veterinary medicines (Regulation (EU) 2019/6) and medicated feed (Regulation (EU) 2019/4) will enter into force within the European Union (EU) from 28 January 2022 [1, 2]. Approved by the European Parliament and Council in late 2018, these regulations include a number of new measures to fight antimicrobial resistance, as outlined in Fig. 1. The regulations also have other objectives. It seeks to promote the availability of veterinary medicinal products by stimulating innovation and competitiveness, to establish a modern, innovative and fit-for-purpose legal framework, and to establish rules applicable throughout the European Union (EU) for the economically viable production of safe medicated feed [2].

These regulations will substantially influence antimicrobial prescribing and usage throughout Europe into the future. Indeed, the impact of these regulations is already being felt in Ireland, including, as one example, the interest in selective dry cow therapy in the Irish dairy industry [3]. These changes should be considered in the context of 2015 data, this being the most recent available, where national coverage of blanket dry cow therapy (DCT) had reached 100% [4]. Blanket DCT is one example of the preventive use of antimicrobials.

These new regulations have been informed by a very large body of work that has been conducted over many years, internationally, by EU agencies, as part of scientific research, and building on relevant earlier policy initiatives by the European Commission. Further, there has been substantial progress towards reduced antimicrobial usage in food animal production in a number of EU member states. This paper seeks to summarise European perspectives on efforts to reduce antimicrobial usage in food animal production.

## International perspectives

Antimicrobials are a critical global resource, and antimicrobial resistance is recognised as one of the most serious current global public health threats [5]. A global action plan on antimicrobial resistance is in place, coordinated by the World Health Organization (WHO) [6], which includes a strategic objective to optimise the use of antimicrobial medicines in humans and animal health. The World Organisation for Animal Health (OIE) has developed the OIE strategy on antimicrobial resistance and the prudent use of antimicrobials [7] in support of this global action plan, and intergovernmental standards on antimicrobial resistance and on the monitoring of the quantities of antimicrobial agents used.

WHO have classified antimicrobials according to their importance for human medicine, as either important, highly important, and critically important antimicrobials. The critically important antimicrobials (CIAs) include those antimicrobials that meet each of the following two criteria: the sole therapy (or one of limited available therapies) to treat serious bacterial infections in people, and a therapy used to treat infection caused by bacteria where there is a potential path for acquisition of resistance, either now or in the future [8]. The CIAs have been further subdivided into high priority and highest priority CIAs based on three prioritisation factors: the number of people treated with infections for which limited antimicrobials are available, the frequency of use in human medicine and among high risk groups, and usage to treat human infections in circumstances where extensive evidence exists about the potential for transmission of resistance bacteria or genes from non-human sources [8]. The highest priority CIAs (HP CIAs) include the quinolones (including fluoroquinolone), 3rd and higher generation cephalosporins, macrolides and ketolides, glycopeptides (such as vancomycin) and polymyxins (for example, colistin) [8].

There has been increasing recognition that widespread antimicrobial usage in food animal production might contribute to the development of resistance to antimicrobials commonly used in human medicine [9, 10], in large part due to the use of common antimicrobials in food-producing animals and humans [11]. The use of HP CIAs in food animal production is viewed with particular concern [10]. For many zoonotic bacteria, the connection between antimicrobial usage and resistance in food animals has clear public health implications. For *Salmonella* spp. and *Campylobacter* spp., the link between antimicrobial resistance in humans and animals is well established, noting that identical mechanisms are used by bacteria from human and animal sources to acquire antimicrobial resistance [12]. For other zoonotic bacteria, including *Escherichia coli*, enterococci and *Staphylococcus aureus*, the human and animal ecosystems are interlinked [12–14]. Collectively, there is now a large body of knowledge of the multiple routes of potential cross-species transmission of antimicrobial resistant genes and bacteria, through food, directly through cross-species contact and indirectly through the environment [11, 12]. It is these One Health concerns that have underpinned policy change, particularly within the EU. For non-zoonotic bacteria, however, there is less clarity about the public health implications of antimicrobial usage and resistance in food animals.

Currently, there are limited quantitative data about the relative impact of antimicrobial usage in livestock for human health. Until recently, quantitative data were also lacking on the benefits for human health of reduced antimicrobial usage in farm animals [15]. This issue was recently addressed in a systematic literature review where Tang et al. [11] found a clear association between antimicrobial usage and resistance in food-producing animals (interventions to restrict usage in food-producing animals was associated with a reduction in the presence of resistant bacteria in these animals). The results also indicate that resistant bacteria can be exchanged between food animals and farm workers, however, evidence is currently weaker and more indirect of transmission to other people.

## The work of the EU agencies

Three EU agencies have focused on antimicrobials and farm animal production, including quantifying antimicrobial usage, reducing antimicrobial usage, and quantifying antimicrobial resistance, including the European Centre for Disease Prevention and Control (ECDC), the European Food Safety Authority (EFSA), and the European Medicines Agency (EMA).

### Quantifying antimicrobial usage

The European Surveillance of Veterinary Antimicrobial Consumption (ESVAC) project was launched within EMA in 2009 following a request from EU member states for harmonised collection and reporting of antimicrobial usage in animals [16]. ESVAC subsequently developed a harmonised approach to data collection and reporting, leading to the publication of detailed usage trends in European countries for 2005–09 [17] and 2010–17 [16]. Usage is based on sales data, and reported as mg/PCU (mg of active ingredient normalised by the population correction unit). PCU is an estimate in kg of the biomass at risk (a proxy for the size of the food-producing animal population, including horses) [18].

In their most recent (2017) report [16], data were available for 31 European countries (all EU member states, Iceland, Norway and Switzerland). Large differences were observed between countries, in terms of mg/PCU, both in sales and in prescribing patterns of various antimicrobial classes. Pharmaceutical forms for group treatments (premixes, oral powders and oral solutions) accounted for 89.4% of all antimicrobial sales, although this varied substantially between countries. Trends on antimicrobial sales during 2010–17, expressed as mg/PCU, are available for 25 of these countries. During this period, there was an overall decline in antimicrobial sales of 32.5%, with some of the largest falls observed in countries where usage had initially been highest. Sales of HP CIAs was low, with a decreasing trend during 2011–17. Specifically, sales of 3rd and 4th generation cephalosporins decreased by 20.9%, polymyxins by 66.4% and fluoroquinolones by 10.3%.

**Fig. 2:**
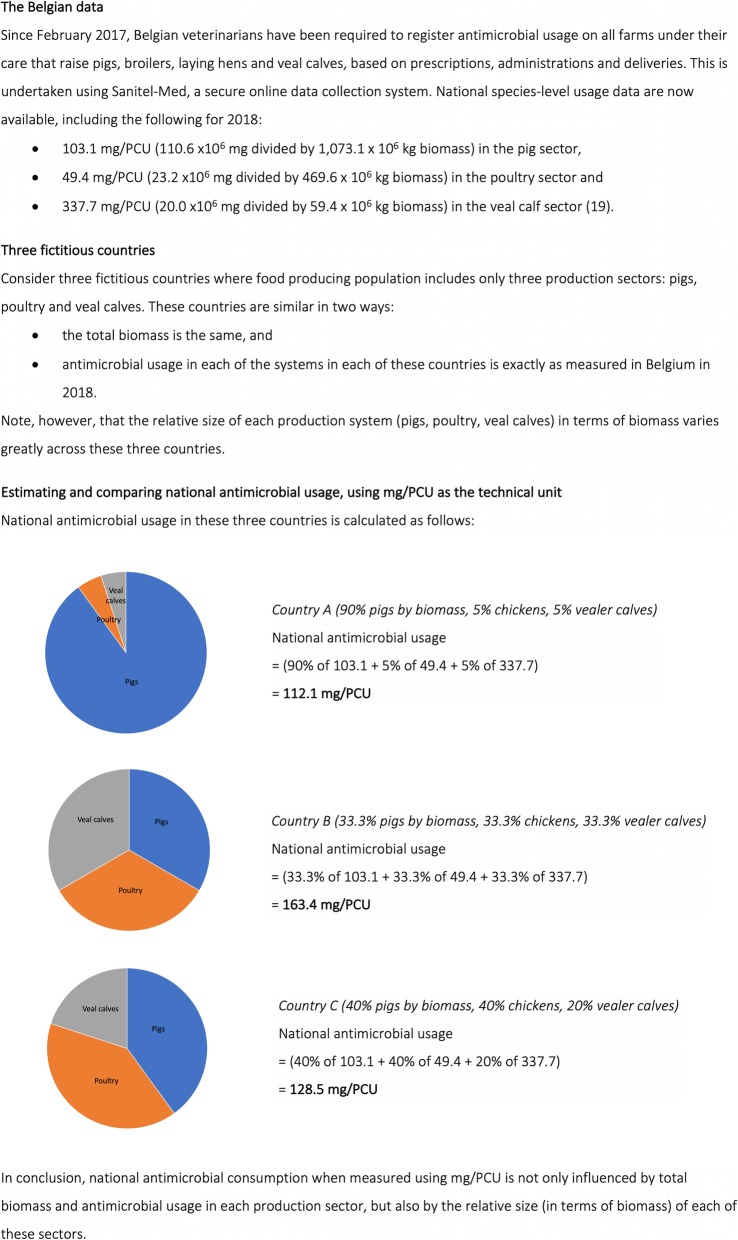
An illustration highlighting the need to interpret country-level differences with care when mg/PCU (population corrected unit) is used as the technical unit. PCU is an estimate in kg of the biomass at risk (a proxy for the size of the food-producing animal population). In the illustration, national antimicrobial usage (in mg/PCU) in three hypothetical countries was very different even though the total biomass and antimicrobial usage within each of three production systems was exactly the same.

Country-level differences need to be interpreted with care when using mg/PCU as the technical unit. Using this unit, national estimates of antimicrobial usage will be influenced by usage in each production system (ie in pigs, in poultry etc), but also by the relative size, in terms of biomass, of each of these systems. Further detail is presented in Fig. 2, using data from a recently released Belgian report [19].

### Reducing the need for antimicrobials

**Fig. 3:**
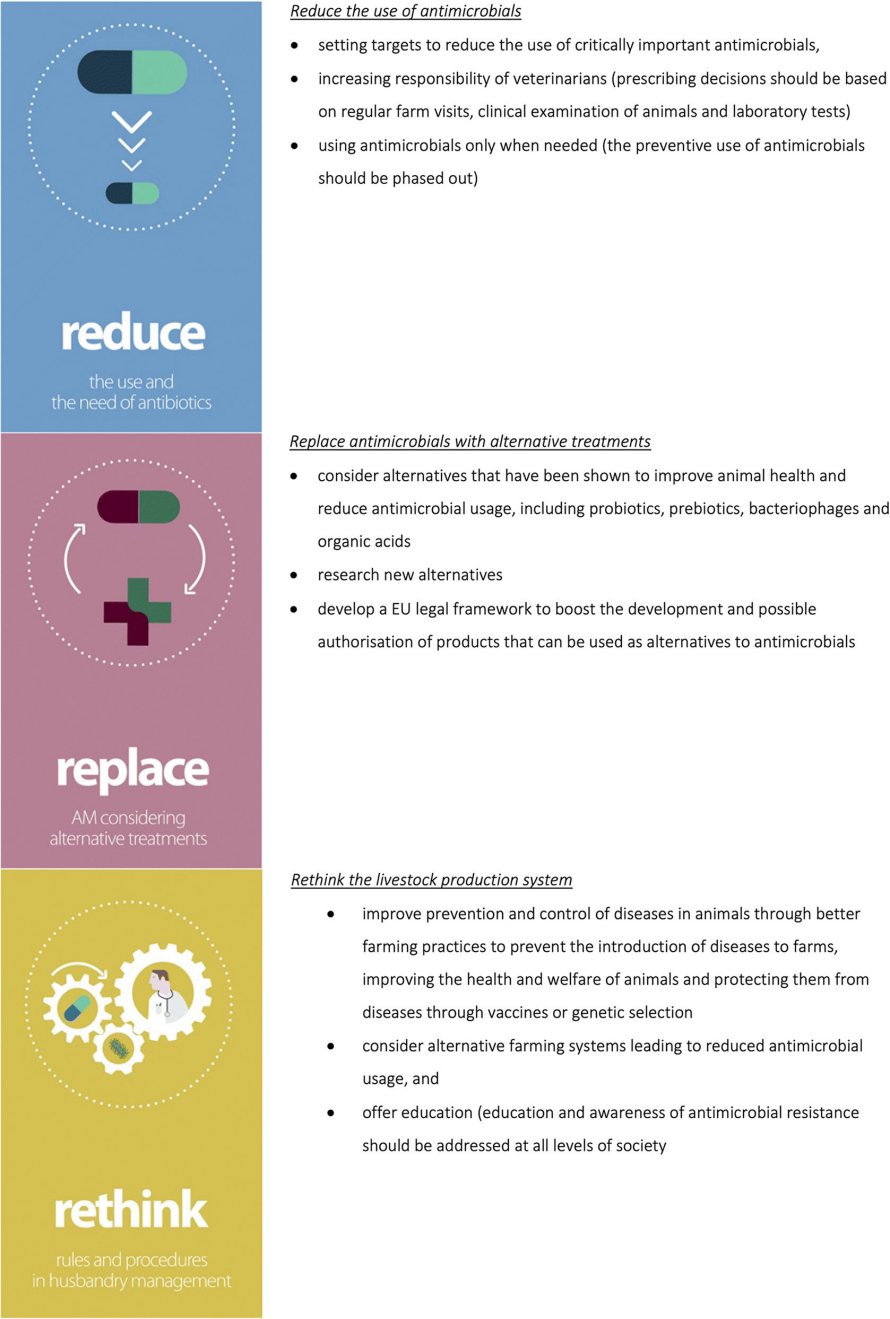
Measures to reduce the need to use antimicrobial agents in food animal production within the European Union. There were the main conclusions from the RONAFA opinion [20], which was published by the European Medicines Agency and the European Food Safety Authority in 2017. The opinion recommends that these measures are addressed within an integrated strategy. The graphics are from EFSA’s interactive infographic ‘How can we reduce the use of antimicrobials in food producing animals?’, (https://www.efsa.europa.eu/en/interactive-pages/Antimicrobial-Resistance) and have been used with permission.

A detailed review, known as ‘the RONAFA opinion’, was published by EMA and EFSA in 2017, to address the need to reduce the need to use antimicrobial agents in animal husbandry within the EU [20]. In part, this work was motivated by the results of the ESVAC project, which highlighted considerable variation in the use of antimicrobials between countries, and also the introduction in some countries of initiatives to successfully reduce antimicrobial consumption. The main conclusions of the RONAFA opinion are presented in Fig. 3.

The opinion recommended that these measures are addressed within an integrated strategy and assessed in terms of effectiveness by monitoring both antimicrobial usage and resistance. Further, it was emphasised that government, industry, health professionals, scientists and consumers all have a role to play [21].

### Quantifying trends in antimicrobial resistance

The EU summary report on antimicrobial resistance in zoonotic and indicator bacteria from humans, animals and food has been produced on an annual basis since 2004, initially by EFSA then jointly by EFSA and ECDC. This work has sought to review data, interpret the findings and assess trends. The work has been strengthened by Commission Implementing Decision 2013/652/EU [22], which outlines a harmonized programme of monitoring for antimicrobial resistance of samples collected from poultry (laying hens, broilers, fattening turkeys), fattening pigs and cattle less than 1 year of age based on susceptibility testing of *Escherichia coli* from caecal samples taken at slaughter, susceptibility testing of *Salmonella* spp. isolates derived from carcase swab samples and testing for the presence of extended-spectrum β-lactamase (ESBL-), AmpC β-lactamase-, or carbapenemase-producing *E. coli* in caecal contents from animals at slaughter and samples of fresh meat at retail [18, 22]. This sampling regime is informed by the emergence in recent decades of a number of plasmid-mediated β-lactamases in Enterobacteriaceae, including *E. coli*. β-lactamases are bacterial enzymes that confer resistance to a variety of β-lactam antimicrobials, including penicillins and cephalosporins [23]. AmpC-producing β-lactamases are intrinsic cephalosporinases of many gram-negative bacteria. Plasmids responsible for ESBL production frequently carry genes coding for other antimicrobial classes; consequently ESBL-producing bacteria are often co-resistant to other antimicrobials including fluoroquinolones, aminoglycosides and trimethoprim-sulphamethoxazole [23]. Carbapenamases are β-lactamases of particular clinical importance, noting that carbapenems are often reserved for the treatment of infections that are caused by otherwise antimicrobial-resistant organisms [24].

The most recent report, based on data collected from 28 EU member states during 2017, highlighted decreasing effectiveness of antimicrobials to treat zoonotic infections, such as campylobacteriosis and salmonellosis [25]. Multidrug resistance (resistance to three or more antimicrobials) is high in *Salmonella* found in humans and animals, particularly in *Salmonella* Typhimurium. Overall, 39.7% of *S.* Typhimurium isolates from people were multidrug resistant, with this percentage varying considerably across member states. In addition, 47.4% of *Salmonella* isolates from pig carcasses were multidrug resistant, most frequently to ampicillin, sulfamethoxazole and tetracycline. High to extremely high proportions of *Campylobacter* isolates from humans were resistant to ciprofloxacin (a fluoroquinolone) and tetracyclines; indeed, resistance in *Campylobacter* to fluoroquinolones was so high in some countries that these antimicrobials are no longer effective for the treatment of severe campylobacteriosis cases in humans. To illustrate, 57.7 and 45.4% of *Campylobacter jejuni* isolates (responsible for > 114,000 reported human cases in 2017) were resistant to ciprofloxacin and tetracyclines, respectively, and in five member states at least 80% of *C. jejuni* isolates were resistant to ciprofloxacin [26].

## Supporting scientific work

### Measuring antimicrobial usage

In recent years, there have been a number of scientific studies of antimicrobial usage in food animal production in Europe. At times, however, different usage indicators have been used (for example, mg/PCU, animal level exposure to antimicrobials [ALEA], defined daily dose for animals [DDDvet], daily dose per 1000 animals [DAPD], treatment incidence based on DDDvet [TI_DDDvet_] etc) which precludes comparison between studies. Collineau et al. [27] recently addressed this issue, providing guidance for the selection of appropriate indicators to quantify antimicrobial usage in food animal production. It is important to note that the selection of appropriate usage indicators depends on their purpose, including whether it is to monitor antimicrobial usage over time (for examples, see the work presented in [28, 29]), to compare usage between different species or countries [30], to allow benchmarking between clinics or farms [29], or to study the association between antimicrobial usage and antimicrobial resistance [28].

Some explanation is needed for the terms DDDvet and ‘defined course dose for animals’ (DCDvet), which are increasingly used as technical units of antimicrobial usage. These units are calculated for a particular animal species and based on the assumed average dose administered per kg per day, noting that a standardised list of DDDvet suitable for use across all EU member states is now available [31]. The following example illustrates the interpretation of DDDvet and DCDvet results, drawing on results reported previously about intramammary antimicrobial usage in the Irish dairy industry [4]. During 2015, the DDDvet for in-lactation usage and the DCDvet for dry cow usage were estimated to be 1398 per 1000 animals per year and 1022 per 1000 animals per year, respectively. Therefore, each cow was treated on average with 1.4 (that is, 1398/1000) in-lactation tubes during the 2015 lactation. Assuming usage as recommended (generally one tube per infected quarter every 12 h on three occasions), this is equivalent to treatment of 466 infected quarters (that is, 1398/3) for every 1000 milking cows during 2015. Similarly, the national coverage of dry cow therapy was just greater than 100% (that is, 1022/1000), noting the assumption that a defined course of dry cow therapy is four tubes per cow, administered at drying off.

### The AACTING consortium

Established in 2017, the AACTING consortium has focused on the quantification of veterinary antimicrobial usage at herd level (AACTING is an abbreviation of ‘network on quantification of veterinary Antimicrobial usage at herd level and Analysis, CommunicaTion and benchmarkING to improve responsible usage’). It has assembled information about existing farm-level systems for the collection of antimicrobial usage data, available at their website [32]. These include systems from a range of European countries and from Canada, and for a number of different farm animal species. In addition, the consortium has developed practical guidelines to support the design of farm-level AMU monitoring systems, with an emphasis on data collection, data analysis, benchmarking and reporting.

### New tools to critically evaluate farm biosecurity

The University of Ghent have developed Biocheck.UGent™, which is an online, freely available, risk-based tool to allow herd- or flock-level biosecurity to be objectively measured [33]. The tool has been developed for use with poultry (layers, broilers), pigs and cattle, and allows external biosecurity (also called bioexclusion; for poultry including purchasing of 1 day old chickens, export of live animals, feed and water supply, removal of manure and dead animals, entrance of visitors and personnel, supply of materials, infrastructure and biological vectors, location of the farm) and internal biosecurity (biocontainment; disease management, cleaning and disinfection, materials and measures between compartments) to be objectively assessed [34]. The tool has been used extensively, both online by individuals, and as part of research projects to quantitatively assess biosecurity (for example: [35, 36]).

## Earlier policy initiatives by the European Commission

The new EU regulations on veterinary medicines and medicated feed have been preceded by a series of earlier relevant policy initiatives. In 2007, the European Commission adopted a new Animal Health Strategy, this being the first time that the Commission had set out its strategic aims and objectives for animal health. With a primary focus on ‘prevention is better than cure’, the strategy was structured around four main pillars (prioritisation of EU intervention; the EU Animal Health framework; prevention, surveillance and preparedness; and science, innovation and research) [37].

The most-recent EU One Health Action Plan against Antimicrobial Resistance was adopted in 2017 [38]. The action plan recognises the connection between human health, animal health and the environment, and particularly emphasises the need for the EU to be a best practice region globally. This and earlier [39] Commission documentation has highlighted the need to boost research, development and innovation in AMR, and for substantially reinforced actions including a regulatory framework for veterinary medicines and medicated feed, and strengthened surveillance systems for AMR and antimicrobial usage in animals.

## Actions by individual member states

Substantial efforts have been made by a number of EU member states to reduce the overall use of antimicrobials in food-producing animals, including the creation of national usage & reduction targets, the measurement and benchmarking of prescribing and usage by veterinary practices and individual farms respectively, and through strategies to encourage antimicrobial stewardship [11]. Actions of individual member states was recently reviewed by O’Neill and Bolton [40].

### Monitoring antimicrobial usage

Since 1996, the Danish Programme for surveillance of antimicrobial consumption and resistance in bacteria from food animals, food and humans (‘the DANMAP project’) has produced a detailed report, produced annually, of antimicrobial usage and resistance in humans and farm animals in Denmark [41]. Summary usage data (at all levels from individual farms through to national) are available by species and production group, and by antimicrobial class. A similar approach has been in place in the Netherlands since 2010 [42], and has now been adopted by a range of other European countries, including Belgium [43], France [44], Sweden [45] and the UK [18]. There are a number of differences between existing monitoring systems for antimicrobial usage, including whether they are government or industry run, by their level of coverage, and by their method of data collection. As one example, recording of antimicrobial usage in Denmark is electronically tied to the billing process [46].

In those countries where national usage data are available, these data are used for multiple purposes including benchmarking of farms and veterinarians and monitoring national and industry-level trends. Using the Netherlands as an example, there is ongoing benchmarking of livestock farms and veterinarians. Several different thresholds (‘signalling and action, representing usage at the 50th and 75th percentile for a defined grouping, such as veal farmers) are used to differentiate between moderate, high and very high users (farmers) and prescribers (veterinarians) [47]. Action is then taken, potentially including disciplinary sanctions, to reduce very high antimicrobial usage and prescribing. Based on similar principles, the yellow card initiative has been operating in Denmark since 2010, to target farms with the highest levels of antimicrobial usage [46, 48]. Since 2016, the differentiated Yellow Card initiative has been introduced to discourage the use of certain critically important antimicrobials. This initiative relies on the use of different multiplication factors for particular antimicrobial classes (including fluoroquinolones, cephalosporins, tetracyclines) to influence overall farm-level usage statistics [49]. In a number of countries, national usage data are available over a series of years, which has allowed objective assessment of temporal trends in antimicrobial usage, both in overall terms, but also by industry and by active compound. This information is critical to the shaping of informed national policy, including an understanding of the impact of different policy initiatives. Limmathurotsakul et al. have recently proposed the concept of the ‘antibiotic footprint’ as a communication tool for the general public, both to increase understanding of the magnitude of antimicrobial consumption by people and in the food animal industries, and also to aid reduction in antimicrobial consumption [50].

In a number of countries, national targets have contributed to a broader strategy to limit antimicrobial usage in food animal production. In Belgium, for example, national 2020 targets include a 50% reduction (compared to 2011) in antimicrobial usage, 75% reduction in CIA usage and 50% reduction in use of medicated feed [51]. Targets may not be evidence-based, but rather based on political imperatives such as the need of the Dutch government to actively respond to growing public demand [47]. Targets can be used as an effective means to motivate change in the food animal industries [20]. In Germany, the introduction of benchmarking alone, without target setting, was also found to be effective in reducing antimicrobial usage [52].

### Restrictions on antimicrobial usage

Several countries have introduced restrictions on antimicrobial prescribing and usage. Further to recommendations from the WHO in 2009 [53], the Netherlands imposed severe restrictions or bans on the specific antimicrobials for food animal use, including 3rd and 4th generation cephalosporins, fluoroquinolones and colistin. The preventive use of all antimicrobials in animals was banned by the Dutch government in 2011 [47]. In Denmark, success in reduction in antimicrobial usage has been attributed to collaboration between the agricultural industry, veterinarians, human health researchers and the government [48].

Recognising the potential for conflicts of interest around antimicrobial usage, several countries have introduced restrictions on veterinarians and farmers, including each of the following. In the Netherlands, farmers are obliged to procure veterinary services and veterinary medicines from a single veterinary practice, to reduce competition between veterinary practices and ensure that the prescribing veterinarian has a comprehensive understanding of the farm [47]. In Denmark, veterinarians have been prohibited since 1995 from profiting from the sale of antimicrobials to their farmer clients [48].

### Additional measures

Consistent with the findings of the RONAFA opinion, a broad range of measures are being used across member states to reduce the need for antimicrobial usage in food animal production.

Farm-level practices were considered in a recent study investigating alternatives to the use of antimicrobial agents in pig production [54]. Drawing on the expertise of more than 100 pig experts in 6 European countries, six strategies were prioritised, based on perceptions of effectiveness, feasibility and return on investment, including biosecurity improvements, increased vaccination, the use of zinc/metals (but noting that the use of veterinary medicinal products containing zinc oxide will no longer be permitted in the EU from June 2022, following an EMA safety and effectiveness review [55]), improvement in feed quality, use of regular diagnostic testing and a clear action plan. This is consistent with the principles of ‘specific pathogen free’ establishments, particularly as applied in pigs and poultry. In recent years, there has been substantial progress in animal breeding towards genetic selection of animals with reduced disease susceptibility [56]. Recent European studies have shown that antimicrobial usage can be reduced concurrent with improved management strategies, with a particular focus on biosecurity, without adversely affecting farm productivity [57, 58] and profitability [59]. Similarly, the withdrawal of HP CIAs did not adversely affect production, health or welfare parameters on UK dairy farms [60]. In many countries, there is an emphasis on communication of best practice to farmers, to improve animal health and thereby reduce the need for antimicrobials. In Ireland, as one example, Animal Health Ireland (AHI; a public:private partnership providing benefits to livestock producers and processors) has developed a wide range of resources for farmers, advisors and veterinarians, including a suite of material to assist with the care of young calves (colostrum management, the use of calf milk replacers, management of scouring calves etc) [61].

At a broader scale, there has been a long history in Europe, and elsewhere, of infectious disease control and prevention in food animal production. These efforts initially focused on regulatory diseases (that is, those of primary concern to government), such as bovine tuberculosis. Increasingly, however, there is increase focus on non-regulatory diseases, such as, for cattle, the control and eradication of bovine viral diarrhoea (BVD), salmonellosis and infectious bovine rhinotracheitis (IBR). This work is frequently coordinated by non-government bodies, such as AHI, Royal GD (also GD Animal Health) in the Netherlands and La Fédération nationale des Groupements de Défense Sanitaire (GDS France), which are playing a key role in coordinating eradication efforts.

Quality assurance (QA) programmes have become increasingly common, offering the potential to positively impact on animal health and antimicrobial usage. Generally independent of government, QA programmes are a direct response to societal and consumer demands for assurance of high standards in animal welfare and food quality [62]. Retailers are playing an increasing prominent role in on-farm antimicrobial stewardship. In the UK, supermarkets have introduced guidelines for antimicrobial usage on supplier farms [63], and farm-level antimicrobial usage data has recently been published [64]. In the Red Tractor Assurance programme, also in the UK, there is considerable emphasis on the responsible use of antimicrobials in the current dairy standard, including the requirement for an annual veterinary review of antimicrobial usage, the use of HP CIAs only as a last resort under veterinary direction, and recommendations for staff training [65]. Nonetheless, some concerns about QA programmes have been raised, relating to the credibility of private animal health and welfare standards within these programmes, the potential use of private standards as a discriminatory barrier to trade, the lack of consumer input in the development of private standards, and the potential (additional) compliance burden placed on farmers [66]. A proposed framework to allow critical evaluation of private animal health and welfare standards in QA programmes has recently been developed [66].

Veterinarians play a central role in the reduction of antimicrobial usage in farm animals. Studies have highlighted major country differences in usage patterns based on sales data [67–69] which in part is linked to cultural, political and societal influences [70]. The challenges facing Dutch veterinarians in their role in seeking to reduce on-farm antimicrobial usage has been considered in some detail [71, 72]. Veterinarians face multiple conflicting interests when making prescribing decisions, which includes the professional obligation to alleviate suffering, financial dependency on clients and risk avoidance [71]. These authors particularly noted the difficulties faced by younger veterinarians in seeking to act independently of the wishes and demands of farmers and others [72]. In the Netherlands, three key challenges were highlighted in seeking to reduce overall use and misuse of antimicrobials in food animals, including the application (successfully and sustainably) of preventive measures on-farm, increased use of appropriate diagnostic tests (preferably pen-side) to guide prescribing decisions, and prudent and accurate administration of antimicrobial treatments [71]. These authors argue that a comprehensive set of interventions (and associated compliance measures) is need to positively influence veterinary prescribing behaviour [71]. Benchmarking of antimicrobial prescribing and use is generally viewed positively by Dutch veterinarians [72]. Detailed treatments guidelines for veterinarians are available in several countries, including Denmark [58]. In the UK, the Responsible Use of Medicines in Agriculture Alliance (RUMA) have formulated comprehensive guidelines for the responsible use of antimicrobials in livestock production, including poultry, pigs, cattle, sheep and fish [73].

## Progress in Ireland

Ireland’s National Action Plan on Antimicrobial Resistance 2017–20 (*i*NAP) provides an overview of Ireland’s commitment to the development and implementation of a holistic, cross-sectoral ‘One Health’ approach to the problem of antimicrobial resistance [74]. Strategic objectives, which mirror those of the WHO’s global action plan to tackle antimicrobial resistance (2015, [6]), include increased awareness and knowledge, enhanced surveillance, reduced spread of infection and disease, optimised use of antibiotics in humans and animals, and promotion of research and sustainable investment.

Relevant to food animal production, a policy on the use of HP CIAs has been developed, indicating that these products should not be used prophylactically or as first line of treatment [75]. Industry stakeholders from the veterinary, farming and pharmaceutical sector have developed a code of good practice regarding the responsible prescribing and use of antibiotics in food animals [76]. The Veterinary Council of Ireland has published guidelines for veterinary practitioners on the ethical use of antimicrobials [77]. There are a number of research projects on antimicrobial usage in food animals in Ireland, relating to pigs [78, 79] and dairy cows [4, 80]. Further, the Biocheck.UGent™ scoring tool has been used to assess biosecurity in the Irish pig and poultry industries. In the dairy industry, AHI has developed guidelines for the use of selective DCT as part of CellCheck, Ireland’s national mastitis control programme [3]. Based on recent evidence (McAloon et al. in preparation), there has been a substantial shift from blanket to selective DCT in the national herd. Finally the Interdepartmental Antimicrobial Resistance Consultative Committee oversaw the publication of Ireland’s first joint One Health Report on Antimicrobial Use and Antimicrobial Resistance, which emphasises the critical contribution of cross-sectoral co-operation to effectively tackle antimicrobial resistance [81].

## Conclusions

In conclusion, this paper highlights some of the work that has been conducted throughout Europe in support of reduced antimicrobial usage in food animal production. In some EU member states, a broad series of changes have been implemented and progress has been substantial. The new regulations offer an important springboard for further progress, in order to preserve the efficacy of existing antimicrobials, which are a critical international resource.

## Data Availability

Not applicable.
